# RF communication between dual band implantable and on body antennas for biotelemetry application

**DOI:** 10.1038/s41598-025-86235-0

**Published:** 2025-02-03

**Authors:** Yara A. Kamel, Hesham A. Mohamed, Hala ELsadek, Hadia M. ELhennawy

**Affiliations:** 1 Microstrip Circuits Department, Electronics Research Institute (ERI), El Nozha, Cairo, 11843 Egypt; 2 Electronics and Communication Engineering Department, Ain Shams University, Cairo, 11566 Egypt

**Keywords:** Biomedical engineering, Electrical and electronic engineering

## Abstract

This paper investigates two antennas for implantable communication, which are a wide-band, low-profile transmitting antenna with a circular polarization (CP) merit immersed in a lossy medium and a corresponding wide-band, low-profile receiving antenna with a linear polarization (LP) merit placed on human tissue. The first antenna is implantable inside a human body for sensing, monitoring, and transmitting various vital signs, while the second antenna acts as a nearby receiving end. These antennas work in the 2.4-2.4835 GHz and 5.725-5.875 GHz industrial, scientific, and medical (ISM) bands. The main features of the designed transmitting antenna are its simplicity, the wide-band characteristics, which preserve the detuning effect caused by environmental heterogeneity, and the CP property at both operational ISM bands. Moreover, for introducing an electrically small antenna footprint with proper performance, the implantable antenna is designed with an entire size of 25.4 (5$\times$ 5 $\times$ 1.016) $\hbox {mm}^3$. This antenna is designed and dissected in a homogeneous skin model (HSM) as well as a three-layer phantom. On the other hand, the wide-band receiving antenna is designed on flexible material for patients’ comfort with a compact size of 134.6 ($20\times 26.5\times 0.254$) $\hbox {mm}^3$. In addition, the implantable antenna performance is evaluated in a chicken slab as well as a saline solution, while the on-body antenna is placed on the chicken slab to measure its reflection coefficient. The measured impedance BWs of the implantable antenna are 13.04 % and 33.2 % in the chicken slab while 19.5% and 25.2 % in the saline solution at the two ISM bands, respectively. While, the measured impedance BWs of the on-body antenna are 24% and 50.4 % at two operating ISM frequencies. Finally, the measured transmission coefficient between the two antennas is evaluated.

## Introduction

Recently, implantable medical devices (IMDs) have gained popularity in the fields of the healthcare industry and medicine^1–3^. This is a result of their ability to transfer pathological or physiological information, such as glucose monitoring, temperature, a leadless pacemaker, blood pressure, etc., from inside the biological tissue to outside facilities^4–7^. This information helps to detect abnormal situations without the need to visit a hospital for examination. In fact, using remote treatment saves patients’ time and reduces the risk of infection^8–11^. Thus, implantable antennas are prime components of IMDs for biotelemetry processes.

As the IMDs have limited space within their a-mm size, the design of the miniaturized antenna has attracted scientific research. However, the miniaturization will affect the implantable antenna performance with regard to efficiency and gain. Thus, many researchers introduced different techniques for achieving compactness without degrading the implantable antenna’s performance^12–16^. Besides, due to the lossy nature of biological tissue and its heterogeneity, where the electrical properties of each human tissue part are different, the detuning effect will occur if the implementation scenario is varied. To avoid the detuning effect, a wide-band antenna is presented in^17^. Another challenge is energy conservation and extending the IMD’s lifetime. In order to address this issue, dual-band antennas were presented in^14,18–20^. In^18^, a dual-band spiral antenna was presented for the WMTS and ISM bands. However, the size of the introduced antenna should be minimized, and the gain value needs to be enhanced. A dual-band linear polarized antenna was presented in^20^. However, this study was concerned with the compactness of the presented antenna size and frequency bands without caring about impedance BW. In^21^, a dual-band antenna with a compact size was introduced for ISM bands. However, narrow impedance BWs were observed. Ultra wide-band linear polarized antennas with a small footprint for the ISM and medical implant communication system (MICS) bands were presented in^22,23^, respectively. However, the dual-band feature was not achieved, which caused power consumption. This is a result of the fact that switching between wake-up and sleep modes wasn’t allowed. Moreover, the linear polarization property of the proposed antennas may be causing a polarization mismatch due to implant orientation through the patient’s movements.

To prevent polarization mismatching of the communication link with the external devices due to biological tissue movement, CP property is an important requirement to alleviate this issue. In^17^, a dual-band patch antenna was introduced for the WMTS band with a CP feature and the ISM band with a linear polarized feature. The designed antenna exhibited a CP BW of 10.3 % at the WMTS band. Besides, the large-size CP antenna was introduced in^24^ and^25^ for the ISM band with a CP BW of 15.8 % and 22 %, and a gain of -33 dBi and -37.36 dBi, respectively. However, minimization of the antennas’ size and enhancement of the gain values were needed. In^26^, a CP antenna working in the ISM band with a gain of -17 dBi was proposed. However, the antenna was operated on a single band with a large size and poor CP BW. Thus, for a good balance between conserving energy, preventing polarization mismatching, and achieving miniaturization, designing a wide-band antenna with a compact size and improved CP property at both operational frequencies is significant. Moreover, enhancing biosensing technology depends on establishing implantable communication with reliable and fast features. The communication link is from transmitting antennas immersed in human tissue to receiving antennas placed outside the patients’ bodies, which transfer the information to the workstation to process it. In contrast to RF communication in free space, implantable communication occurs through biological tissue, which is a hostile channel, causing the attenuation of the EM field strength, especially for high frequencies. Thus, the on-body antennas retransmit the received weak signals and assist in decreasing the needed IMDs’ power level, resulting in a reduction of the absorbed EM waves and saving the battery’s lifetime.

In this study, implantable Tx antenna and on-body Rx antenna are proposed for bio-telemetry applications working in the 2.41 and 5.81 GHz ISM bands. A low-profile dual-band CP implantable patch antenna with a wide BW is designed to be suitable for placing inside the patient’s tissue. The proposed dual-band CP implantable patch antenna is studied in a three-layer phantom as well as HSM. The miniaturization of the proposed antenna and enhancement of its impedance matching at both operational ISM frequencies are carried out by loading the via between the ground plane and the radiating patch antenna, loading meander lines at the edge of the central hexagonal slot, etching two rectangular slits at the edges of the radiator, and loading a T-shaped ground plane. Furthermore, as the two rectangular slits at the boundary of the radiating patch antenna and T-shaped ground plane are adjusted, the CP at two bands is achieved. While, a flexible on-body antenna with a compact size and wide band is designed for patient comfort. The designed antenna begins with a conventional patch antenna that has a partial ground plane. However, in order to achieve a low profile and improve impedance matching, a center slot is loaded on the radiator to increase the capacitive effect. Furthermore, two meander lines are etched to expand the current path for shifting the frequency down and enhancing the impedance matching. Then, a parasitic element is etched on the ground plane for operating at desired frequencies.

The designed transmitting antenna is compared to the performance of recent literature implantable patch antennas that are immersed in the same surrounding tissue, as visualized in Table 1. As illustrated from the comparison, larger-sized implantable antennas than our designed antenna are introduced in^17,18,24–27^. Although the proposed antennas in^20,21^ were smaller in size, the impedance BWs were narrow. In^22,23^, the designed antennas exhibited ultra-wideband performance and compact size. However, the single-band feature and linear polarization of the designed antennas may be causing power consumption and polarization mismatching, respectively. Besides, the designed antenna is preferable for operating at dual-band CP than the literature antennas, which are designed for either a single band of operation with a CP feature as in^24–27^ or dual-band antennas with linear polarization as in^18,20,21^ and with a CP feature at a single band as in^17^. The other merits of the designed antenna are demonstrated in Table 1.

**Table 1:** Proposed antenna compared to recent literature antennas.

Ref.	Volume	Dep.	Freq.	BW	ARBW
	($$mm^3$$)	(mm)	(GHz)		
^17^	103.7	3	1.4		10.38%
			2.45	21.3%	–
^18^	154.88	3	1.42	3.1%	–
			2.45	6.1%	
^20^	64.65	2	0.4	5.7%	–
			2.45	7.7%	–
^21^	24	4	0.915	9.8%	–
			2.45	8.5%	–
^22^	9.8	12	2.45	42.3%	–
^23^	79.4	–	0.402	33%	–
^24^	121.9	3	2.45	12.5%	15.8%
^25^	63.8	4	2.4	16.6%	22%
^26^	91.76	2	2.45	12.2 %	2.4 %
^27^	121	4	2.45	8.3%	2.49%
This work	25.4	2	2.41	16.8%	8.3%
			5.81	22.5%	18.8%

## Design methodology

### Implantable Tx antenna design

**Fig. 1:**
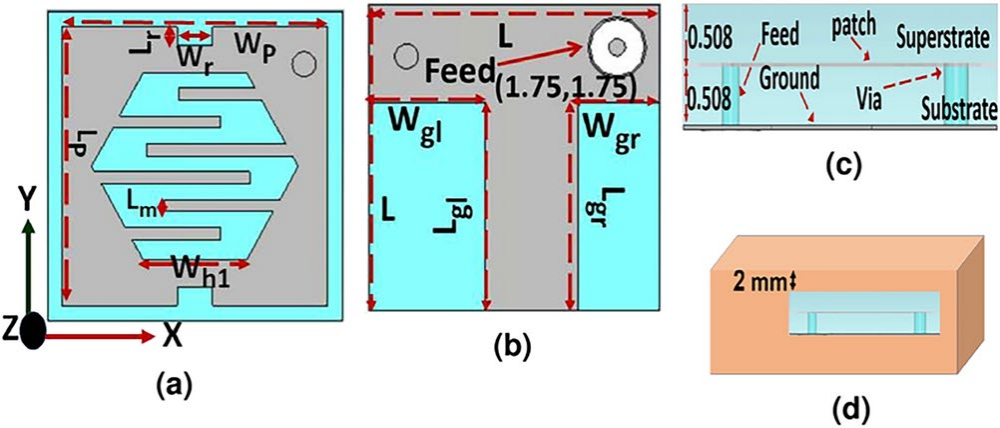
Antenna configuration (unit:mm) (**a**) radiator geometry with dimensions $\hbox {W}_{P}=\hbox {L}_{P}=$4.5, $\hbox {W}_r=$0.6, $\hbox {L}_r=$0.3, $\hbox {L}_m=$0.2, $\hbox {W}_{h1}=$1.75, (**b**) ground plane geometry with a dimensions of L$=$5, $\hbox {W}_{gl}=$2, $\hbox {W}_{gr}=$1.4, $\hbox {L}_{gl}=$3.4, $\hbox {W}_{gr}=$3.4, (**c**) side view, and (**d**) Homogeneous skin model.

Figure 1a–c visualize the configuration of the compactly dual-band CP implantable patch antenna. The dimensions of the designed antenna are 5$\times$5$\times$1.016 $\hbox {mm}^3$. The Fig. 1a exhibits the radiating element, the T-shaped ground plane is depicted in Fig. 1b. While the side view of the compactly designed antenna is exhibited in Fig. 1c. To condense the size of the proposed antenna, the center hexagonal slot with six meander lines at its boundary is etched on the radiating element. These meandering lines are used to lengthen the current flow. Besides, the shorting pin is located between the square radiating element and the ground plane at a certain appropriate position. To realize CP excitation, two rectangular slits are etched in opposite directions. While the T-shaped ground plane helps in downsizing the designed antenna and enhancing impedance matching and bandwidth. The diameters of 0.28 and 0.4 mm are chosen for 50-$\Omega$ coaxial feed cable and via, respectively.

The antenna is fabricated using a 0.508 mm thick Roger RO3010 with tangent loss tan($\delta$)= 0.0027 and $\varepsilon _{r}$= 10.2. This material is also employed as a covered layer to avoid direct touch with lossy biological medium. The dimensions of the antenna are optimized using Computer Simulation Technology (CST).

In contrast to free-space antennas, the implantable antenna is designed in the HSM with a size of (130$\times$ 130$\times$ 45.27)$\hbox {mm}^3$ at an implementation depth of 2 mm, as visualized in Fig. 1d^28^. As the electrical characteristics of the simulation environment are frequency-dependent, the complex permittivity is evaluated using the formulation of the Cole-Cole model as^29^

1 $$\begin{aligned} \varepsilon _r(\omega )=\varepsilon _{\infty }+\sum _{n}\frac{\Delta \varepsilon _n }{1+(j\omega \tau )^(1-\alpha _{n})} +\frac{\sigma _{i}}{j \omega \varepsilon _0} \end{aligned}$$

where $\varepsilon _{\infty }$ represents the relative permittivity of the high frequency, $\tau$ represents the relaxation time, $\Delta \varepsilon _n$ represents the pole amplitude, and *n* denotes the poles number. The parameters value are $\Delta \varepsilon _1=32$, $\sigma =0.0002$, $\alpha _{2}=0.2$, $\Delta \varepsilon _4=0$, $\Delta \varepsilon _3=0$, $\varepsilon _{\infty }=4$, $\alpha _{1}=0$, $\tau _2= 32.48 (ns)$, $\Delta \varepsilon _2=1100$, $\tau _1=7.23 (ps)$.

### Design stages of implantable antenna

**Fig. 2:**
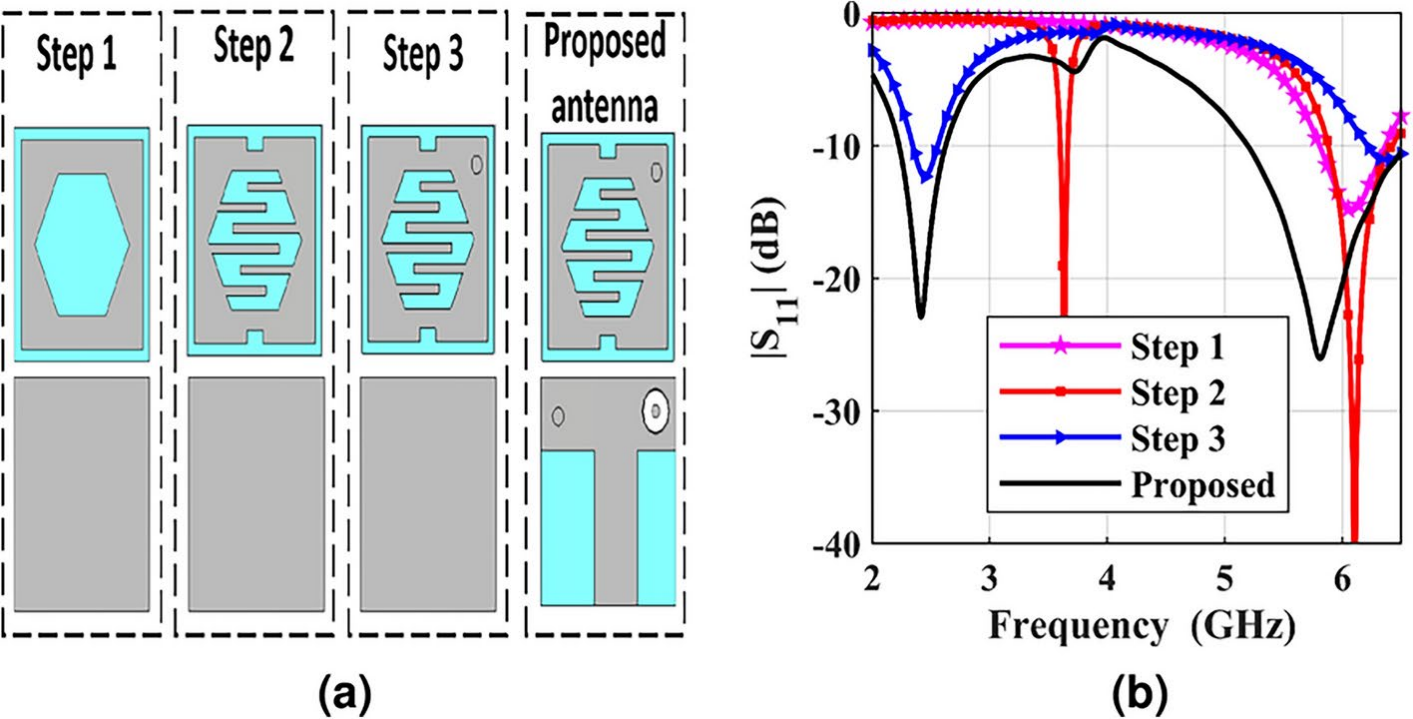
(**a**) Evolution stages and (**b**) accompanied antenna behavior.

The miniaturization process of the dual-band CP designed antenna is performed in four steps, along with its reflection coefficient, as demonstrated in Fig. 2. Initially, the dimension of the conventional square radiating patch is given by^30,31^

2 $$\begin{aligned} f_r=\frac{c}{\lambda _g \sqrt{\varepsilon _{eff}}}\approx \frac{c}{L_g \sqrt{\frac{\varepsilon _r+1}{2}}} \end{aligned}$$

where $f_r$ represents the operating frequency, $L_g$ denotes the effective length, $\lambda _g$ denotes the guided wavelength, $\varepsilon _r$ represents the relative permittivity, and $\varepsilon _{eff}$ represents the effective permittivity. In step 1, the central hexagonal slot is etched on the square radiating patch to lengthen the current flow and increase the capacitive effect. The capacitance of the designed antenna is increased with decreasing propagation velocity $v_p$ according to Eq. 3, thus the miniaturization is carried out.

3 $$\begin{aligned} v_p=\frac{1}{\sqrt{L_0 C_0}}=\frac{c}{\sqrt{\varepsilon _{eff}}}=\lambda _g f \end{aligned}$$

In step 2, the meander lines are loaded at the edge of the central hexagonal slot, and two rectangular slits are printed in the opposite direction on the radiating patch antenna to assist in increasing the current path. It’s observed that the operating frequency of the dual-band CP implantable antenna is moved from 6.086 to 3.69 GHz, whereas the other operating frequency is 6.104 GHz. Besides, as the probe feed is inserted along the diagonal of the square radiating patch and two rectangular slits are etched on its edges, the CP property is excited. The shorting pin is embedded in the antenna’s substrate at the right corner of the square radiating element, down to the ground plane, which results in shifting the operating frequency down in step 3. For detuning the desired frequencies, enhancing the impedance matching, and improving the bandwidth, T-shaped ground plane is loaded in step 4. Besides, CP performance is achieved. As visualized in Fig. 2b, the BWs of the introduced antenna are 16.8% (2.222-2.629) GHz at 2.41 GHz, and 22.5% (5.19-6.5) GHz at 5.81GHz.

### Effect of three-layer phantom

**Fig. 3:**
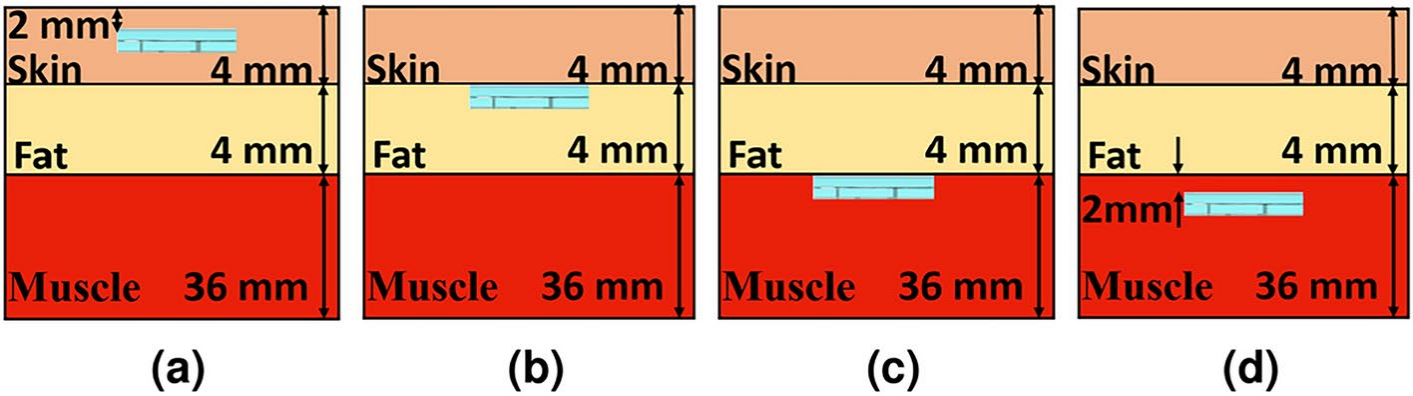
Three-layer phantom with four different cases.

**Fig. 4:**
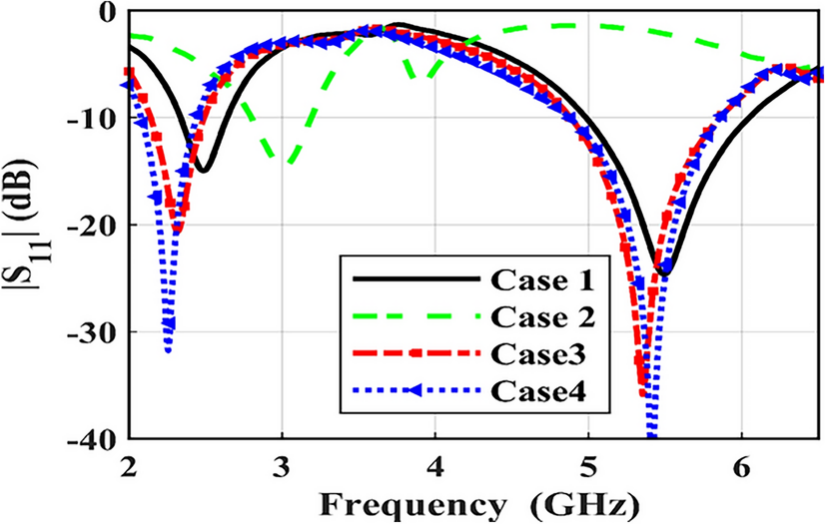
S-parameters comparison for four different cases.

The sensitivity of the designed dual-band CP implantable antenna is evaluated by immersing it in another simulation model (body phantom) to ensure its availability for implanting it in different positions and using it in other bio-telemetry applications. The designated antenna is implanted in a three-layer phantom, which consists of skin, fat, and muscle layers, with dielectric properties mimicking those of human tissue, as visualized in Fig. 3. The electrical characteristics of these layers are listed in^32^. As observed in Fig. 4, the antenna performance in skin and muscle cases has small changes. The desired frequencies are shifted lower in the muscle case than in skin implantation because of its higher permittivity. However, the antenna performance changes significantly in the fat implementation compared to the other cases. This is due to the lower relative permittivity of this layer, which shifts the desired frequencies up.

### CP realization

**Fig. 5:**
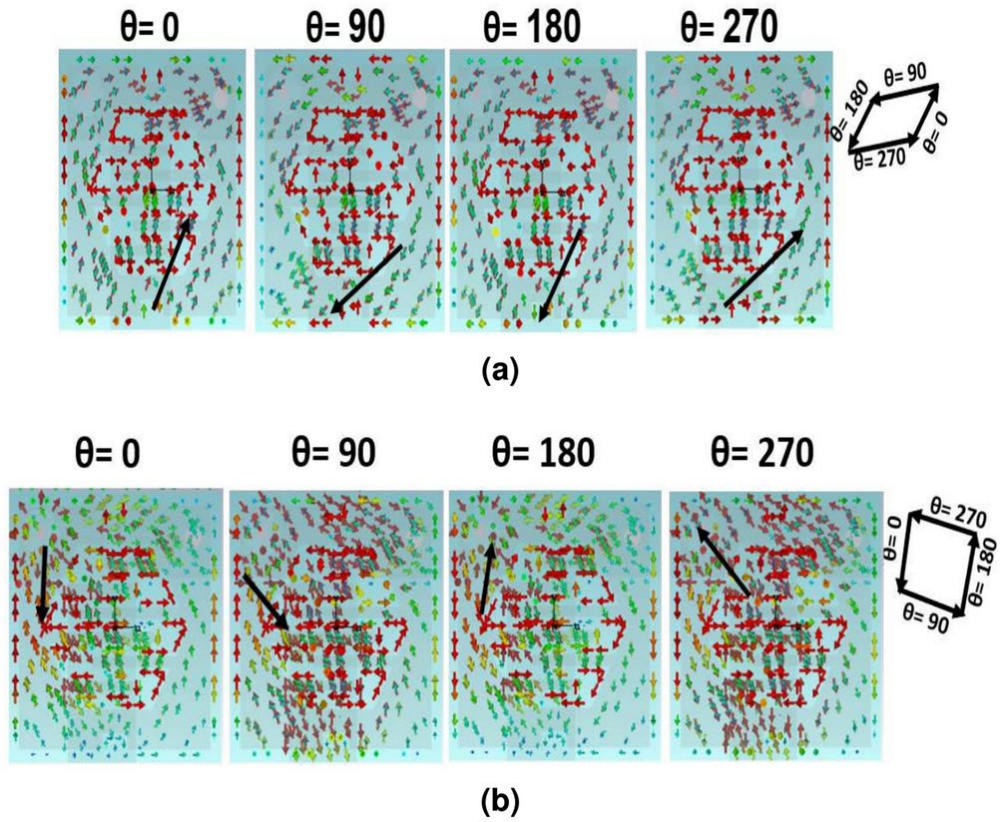
Current distribution on the radiating patch antenna for different phases at (**a**) 2.41 GHz and (**b**) 5.81 GHz.

**Fig. 6:**
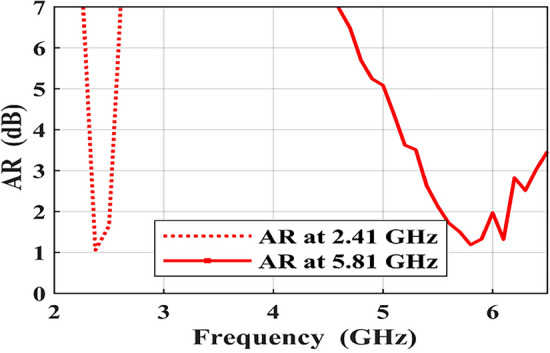
AR of the introduced implantable patch antenna.

A shorter path is created along the y-direction as a result of inserting two rectangular slits, which cause the dominant mode to divide into two degenerate orthogonal modes with $\hbox {90}^\circ$ phase difference and equal amplitude. Besides, the T-shaped ground plane introduced a perturbation by introducing the two different paths of the current flow, which excites the CP property. Thus, the behavior of the current distribution on the square radiating element with respect to different phases ($\hbox {0}^\circ$-$\hbox {270}^\circ$) at the two desired ISM bands is depicted in Fig. 5a and b, respectively. As shown,

most dominant current vectors on the upper right direction of the square radiating element at $\hbox {0}^\circ$ phase and reversed its direction with the same path at $\hbox {180}^\circ$. While at the $\hbox {90}^\circ$ phase, the current vectors to the lower left direction and reversed its tendency with the same path at $\hbox {270}^\circ$. The direction of the combined current, which is demonstrated by the black arrows, rotates anticlockwise. This emphasizes that the polarization sense in the lower ISM band is right-hand CP (RHCP). At the higher ISM band, the most dominant current flowed on the lower side of the square radiating element at $\hbox {0}^\circ$ phase and reversed its direction with the same path at $\hbox {180}^\circ$. While at the $\hbox {90}^\circ$ phase, the current flowed to the lower right side and reversed its direction with the same path at $\hbox {270}^\circ$. The direction of the combined current, which is demonstrated by the black arrows, rotates anticlockwise for different phases. This emphasizes that the polarization sense in the higher ISM band is right-hand CP (RHCP). Also, the 3-dB AR BWs are being investigated to characterize the CP behavior at both operational frequencies, as demonstrated in Fig. 6. As observed, the 3-dB AR BWs at the lower 2.41 GHz and upper 5.81 GHz ISM bands are 8.3% (2.3-2.5) GHz and 18.8% (5.3-6.4) GHz, respectively.

### Safety consideration

**Fig. 7:**
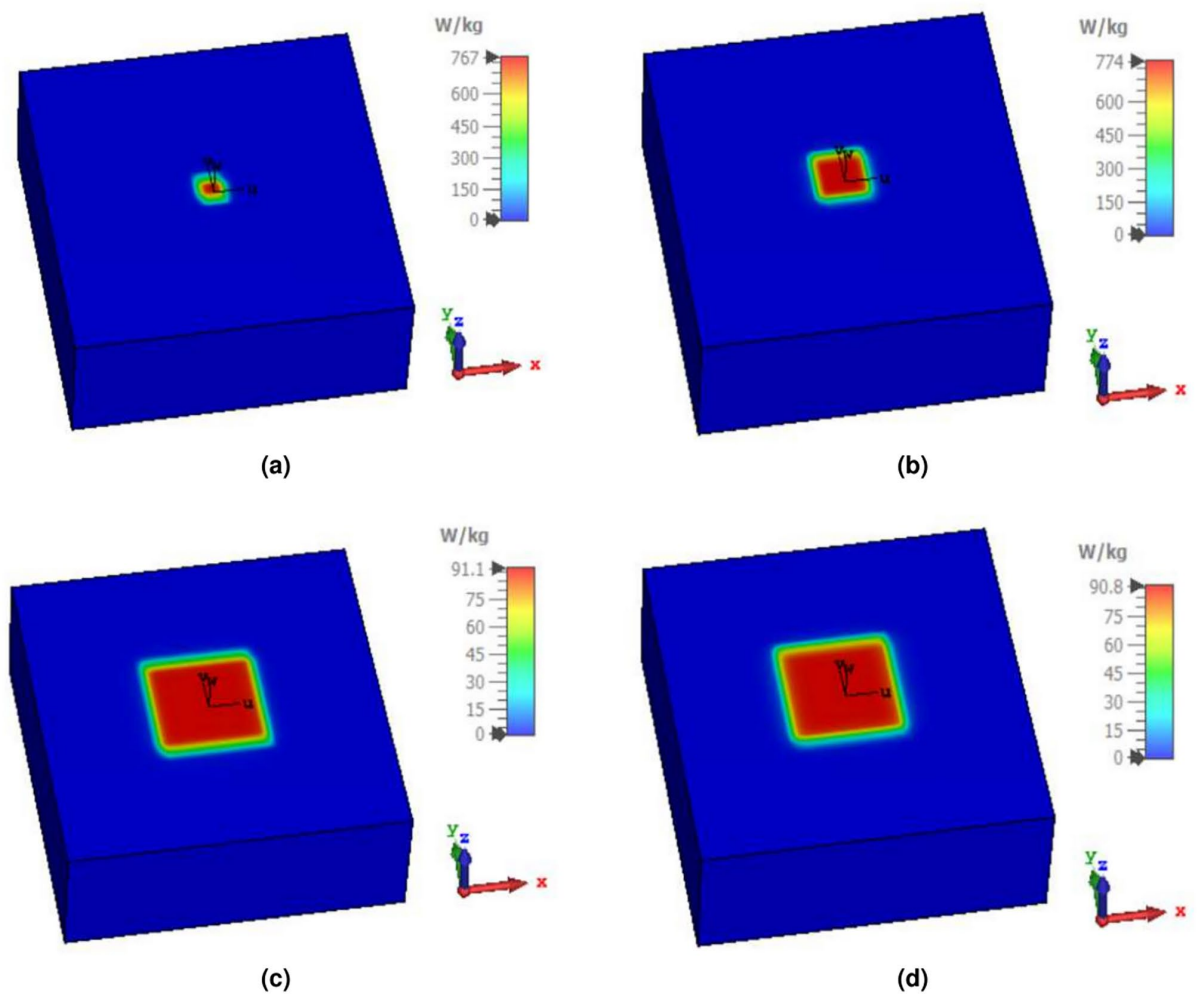
Maximum averaged values of SAR for (**a**) 1-g at 2.41 GHz, (**b**) 1-g at 5.81 GHz, (**c**) 10-g at 2.41 GHz, and (**d**) 10-g at 5.81 GHz.

As the biological tissue is exposed to electromagnetic (EM) waves during the wireless biotelemetry process, patient safety is a crucial factor in the design of the antenna. Thus, SAR is evaluated according to IEEE C95.1-1999 and C95.1-2005 standards, which restrict the averaged SAR for 1-g and 10-g of the cubic biological tissue to 1.6 W/Kg and 2 W/Kg, respectively^33–35^. By delivering the designed antenna with 1 W of input power, the averaged SAR values are 767W/Kg and 774W/Kg over 1-g, as depicted in Fig. 7a and b. The associated input power should be below the values of 2.08 and 2.06 mW at lower and higher operating ISM frequencies, to meet the IEEE C95.1-1999 restriction. Furthermore, the simulated averaged SAR values with the 1 W input power excitation are 91.1W/Kg and 90.8W/Kg over 10-g , as demonstrated in Fig. 7c and d . To meet the IEEE C95.1-2005 restrictions, the input power delivered to the implantable antenna should be below 21.9 and 22.02 mW at 2.41 GHz and 5.81 GHz, respectively. As the allowed input power stated by the European Research Council (ERC) is much lower than the maximum input power, this indicates that the designated antenna doesn’t have a serious concern on the patient safety.

### On-body Rx antenna design

**Fig. 8:**
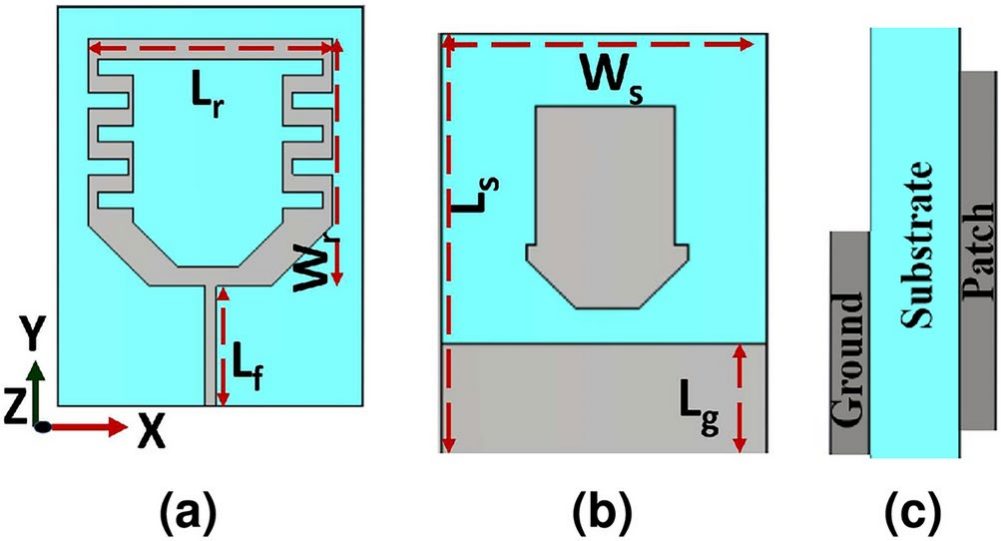
Designed on-body antenna geometry (unit:mm) (**a**) front view, (**b**) back view, and (**c**) side view.

The structure of the on-body antenna with a low profile is shown in Fig. 8. The physical dimensions of the optimized antenna are 20 $\times$ 26.5 $\times$ 0.254 $\hbox {mm}^3$. The radiator of the designed antenna is depicted in Fig 8a. For shifting the frequency down, the central slot and two meander lines are etched on the patch antenna. In Fig. 8b the partial ground plane and parasitic element are introduced on the other substrate side. While the side view of the on-body antenna is demonstrated in Fig. 8c.

The designed antenna is excited by a 50$\Omega$ microstrip line. For the patient’s comfort, the antenna is printed on a flexible substrate Roger RO5880 with a relative permittivity $\varepsilon _r=$ 2.2, loss tangent $tan(\delta )=$0.0009, and 0.254 mm thick. The dimensions of the on-body antenna are optimized on Computer Simulation Technology (CST) as listed: $L_{s}$=26.5, $W_{s}$=20, $L_{r}$=18, $W_{r}$=16, $L_{g}$=8, $L_{f}$=8

### Design evolution of the on-body Rx antenna

**Fig. 9:**
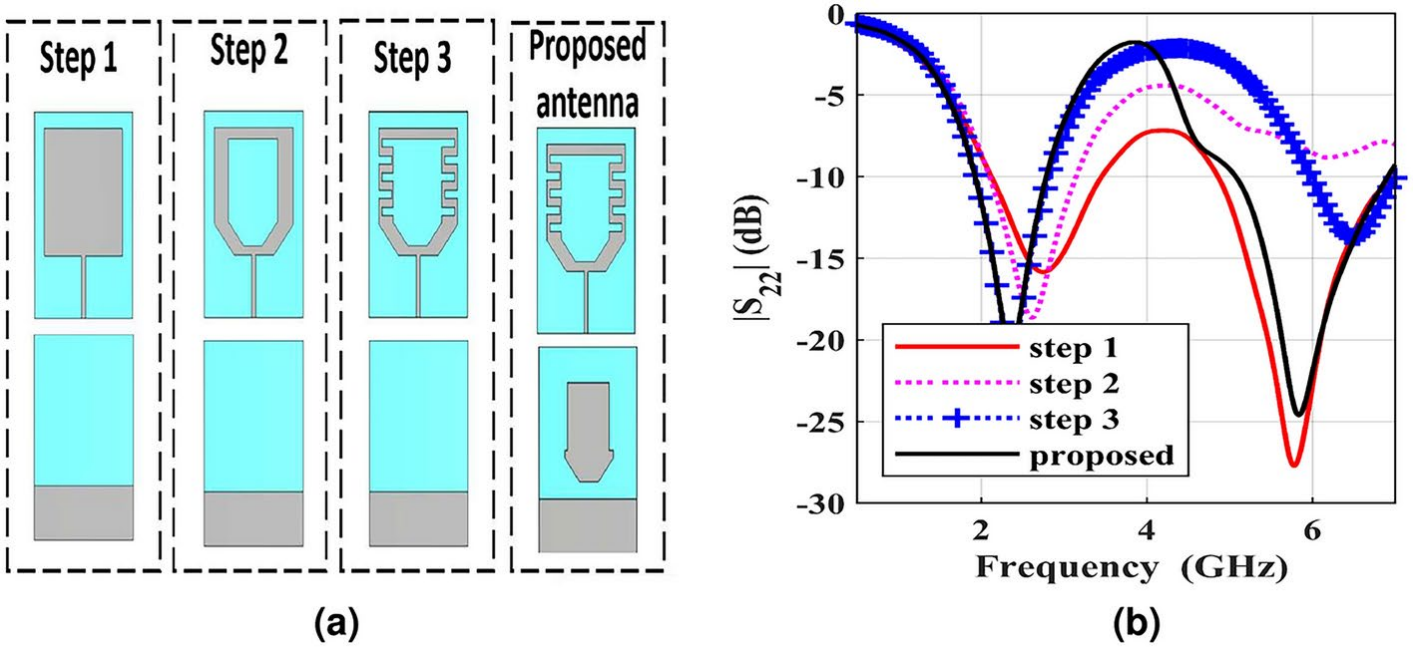
(**a**) On-body designated antenna evolution, and (**b**) the reflection coefficient at each stage.

The optimization of the designated on-body antenna is achieved in four steps, as depicted in Fig. 9a. Initially, the evolution of the optimized antenna on the human body is based on the conventional partial ground plane microstrip antenna. As the antenna in this work is worn on the patient’s tissue, the human body can act as the antenna’s load. Thus, the design of the wearable antenna is estimated by Eq. 2. In step 2, the patch antenna’s lower edges are chamfered, and a center slot is loaded to increase the capacitive effect, which helps shift the frequency down from 2.76 GHz to 2.61 GHz in the lower ISM band. However, it’s causing poor impedance matching in the higher ISM band. Thus, by printing two meander lines at the right and left edges of the radiating element, the current expansion occurs, causing the lower operating frequency to shift from 2.61 GHz to 2.4 GHz and enhancing the impedance matching of the higher ISM band in step 3. In step 4, the parasitic element is added on the other side of the substrate to make a slight improvement in the impedance matching of the lower ISM band and shift the upper ISM band from 6.4 GHz to 5.8 GHz. As observed in Fig. 9b, the impedance BW of the proposed antenna on the three-layer model at lower 2.41 GHz and upper 5.81 GHz ISM bands are 33 % (1.8-2.6) GHz and 34.4 % (4.8-6.8)GHz, respectively.

### Design of implantable communication

**Fig. 10:**
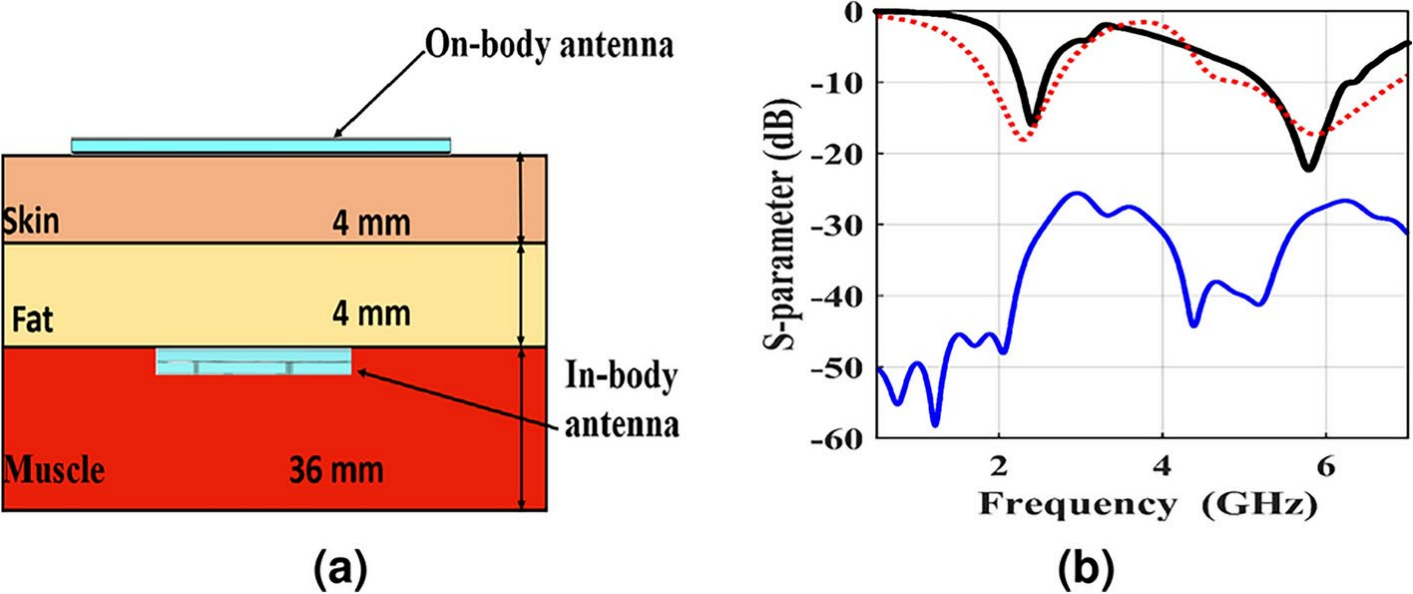
(**a**) Scenario of implantable communication in the simulation environment, and (**b**) simulated reflection coefficient ($|S_{11}|$) of the Tx antenna (Black line) , Rx antenna ($|S_{22}|$) (Red dotted line) and transmission coefficient ($|S_{21}|$) (Blue line).

The requirements of communication performance take the effect of human tissue into consideration. The arrangement setup of the implantable communication between two antennas is illustrated in Fig. 10a. As shown, the implantable Tx antenna from section 1.1 is immersed in a muscle layer, and the on-body Rx antenna from section 1.6 is in direct contact with the skin layer of the three-layer phantom, which is in the near-field region. Thus, the far-field communication evidence, such as radiation pattern and gain, are excluded in this work. The performance of the system is analyzed based on S-parameters, i.e., the transmission coefficient between two antennas ($|S_{21}|$), the reflection coefficient of the transmitting antenna ($|S_{11}|$), and the reflection coefficient of the receiving antenna ($|S_{22}|$), as demonstrated in Fig. 10b. As illustrated, the transmission coefficients are -32 dB and -28.3 dB at 2.41GHz and 5.81GHz, respectively. The reflection coefficient illustrates that two antennas operate at lower and higher ISM bands. The determination of the power transmission across the RF link can be determined by squaring $|S_{21}|$ ($|S_{21}|^2=P_{r}/P_{t}$) where $P_r$ is the delivered power to the Rx and $P_t$ is the Tx available power. As a result, the equation of the link is given by^31^

4 $$\begin{aligned} |S_{21}|^2=\frac{G_{t} G_{r} \lambda _0^2}{4 \pi r^2} (1-|S_{11}|^2)(1-|S_{22}|^2)e_{p} \end{aligned}$$

where $G_{t}$ represents the gain of the implantable Tx antenna, $G_{r}$ denotes the gain of the on-body Rx antenna, *r* represents the distance between the implantable and on-body antennas, and $e_{p}$ represents the factor of the polarization mismatch.

Besides, the power flow is simulated to visualize the operating principle of the system. The Poynting vector defines the power in the EM field per unit area as^31^

5 $$\begin{aligned} S=\frac{1}{2} {\textbf {E}} \times {\textbf {H}}^{*} \end{aligned}$$

where **H** and **E** denotes the auxiliary magnetic field and electric field, respectively. Hence the power flowing can be evaluated as

6 $$\begin{aligned} P=\oint S. ds \end{aligned}$$

**Fig. 11:**
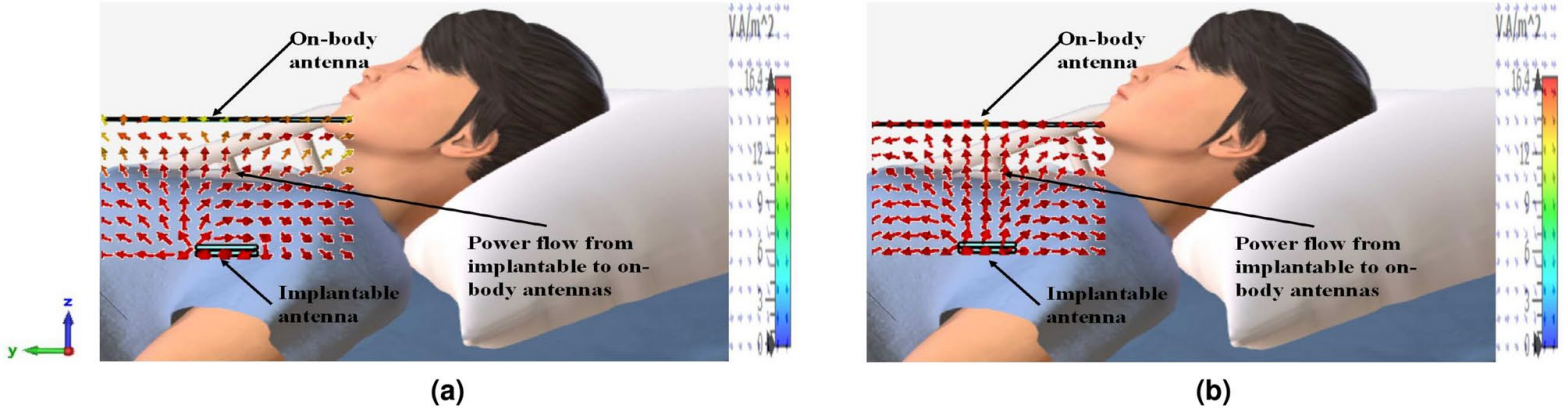
The distribution of the Poynting vector at (**a**) 2.41 GHz, and (**b**) 5.81 GHz.

The distribution of the poynting vector at 2.41 GHz and 5.81 GHz is demonstrated in Fig. 11. As observed, the electromagnetic (EM) wave is strongly distributed in the on-body Rx antenna direction, which is located outside the biological tissue.

## Experimental verification and discussion

To verify the simulated findings of the introduced dual-band implantable and on-body antennas, the prototype antennas are fabricated on the upper and lower planes of the substrates Rogers RO3010 and RO5880, respectively. The proposed antennas are fabricated using the photo-lithographic method.

**Fig. 12:**
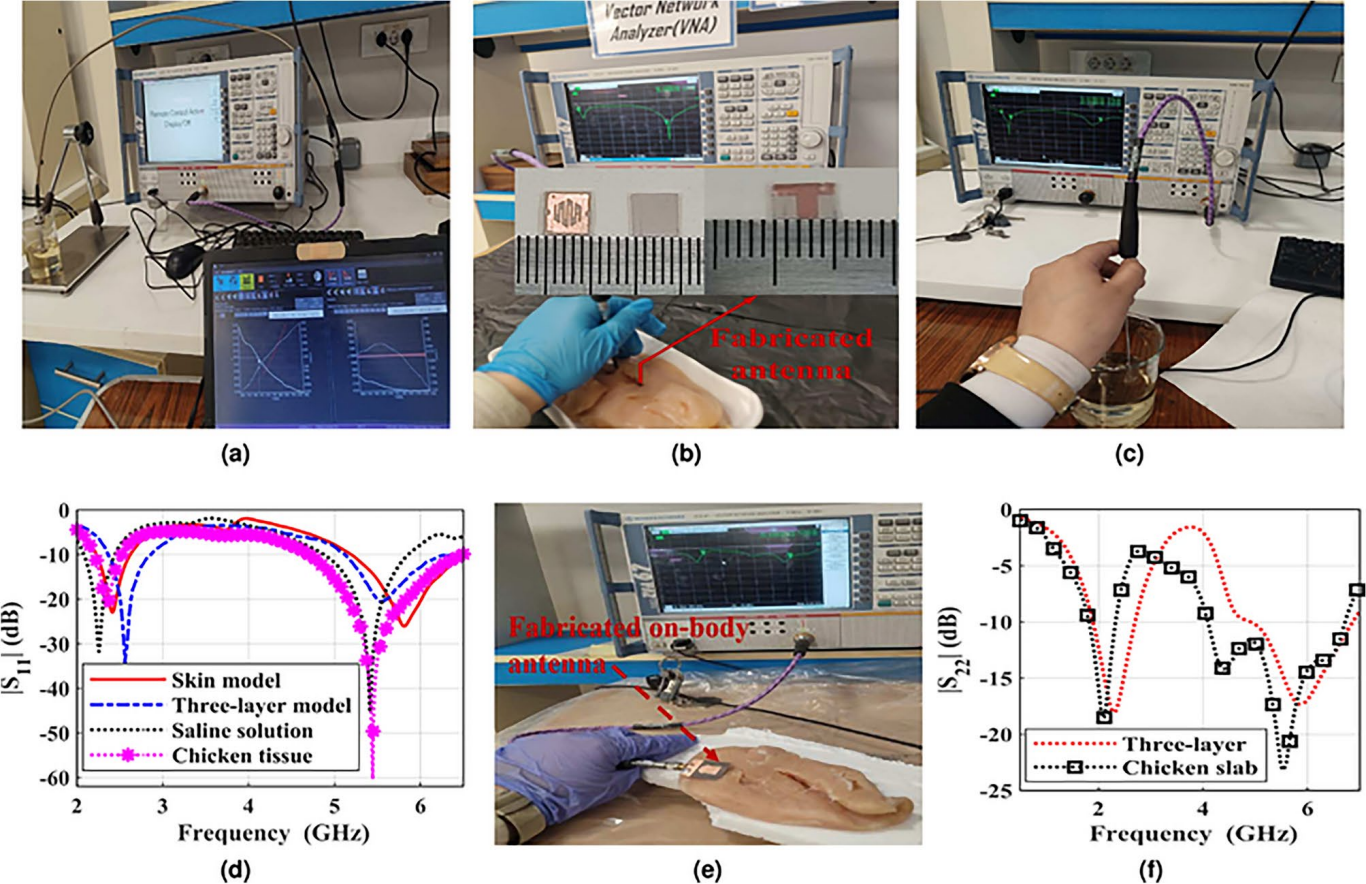
(**a**) Measurement setup of the saline solution dielectric properties, (**b**) measurement setup of the introduced implantable antenna in chicken tissue, (**c**) measurement setup of the introduced implantable antenna in solution, (**d**) comparison between measurements and simulation results of the implantable antenna, (**e**) measurement setup of the on-body antenna on chicken tissue, and (**f**) comparison between measurements and simulation results of the on-body antenna.

The ZVA67 vector network analyzer (VNA) is connected to the fabricated implantable and on-body antennas through coaxial cable and SMA connector, respectively. The saline solution is prepared with portions and ingredients of 53 % water and 47 % suger. Then the SPEAGE dielectric assessment kit (DAK) is used to measure the electrical characteristics of the saline solution as demonstrated in Fig. 12a. For measuring the reflection coefficient ($|S_{11}|$), the fabricated implantable antenna is implanted into a chicken slab, which was purchased from a chicken vendor, as well as a saline solution, as demonstrated in Fig. 12b and c, respectively. As observed in Fig. 12d, the measured BWs of the printed antenna in the chicken slab are 13.04% (2.2-2.5) and 33.2% (4.69-6.5) GHz at the lower 2.41 GHz and upper 5.81 GHz of the ISM band, respectively. While the measured impedance BWs when placing the antenna into saline solution are 19.5% (2.36-2.86) and 25.2% (5.1-6.5) GHz at lower and higher operating ISM frequencies, respectively. On the other hand, the fabricated on-body antenna is placed on a chicken slab for measuring the reflection coefficient ($|S_{22}|$), as depicted in Fig. 12e. As demonstrated in Fig. 12f, the measured BWs when the fabricated antenna is placed on the chicken slab are 24% (1.8-2.3) GHz and 50.4% (4-6.7) GHz at two operating ISM frequencies.

**Fig. 13:**
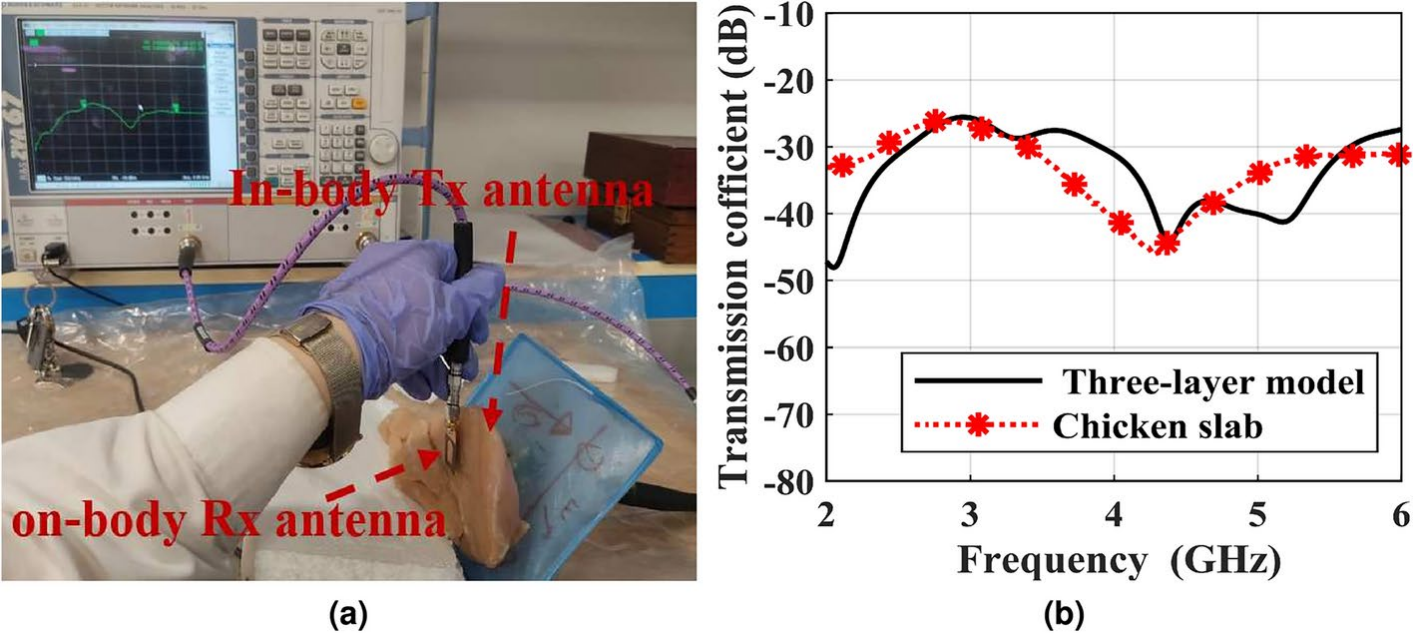
(**a**) Measurement setup of the transmission coefficient, and (**b**) comparison between measurements and simulation results of the transmission coefficient.

The implantable communication between the implantable Tx antenna and the on-body Rx antenna is depicted in Fig. 13a. As observed, the implantable antenna is immersed in the chicken slab while the on-body antenna is located on it in the near-field region. For measuring the transmission coefficient , the Tx antenna is connected to port 2, and the Rx antenna is connected to port 1 of the VNA. A comparison between measured and simulated transmission coefficients is demonstrated in Fig. 13b. As detected from the figure, the little difference between the measured and simulated results might be a result of different transfer distances, the soldering of the SMA and coaxial cable, and the difference between the simulated and measurement mediums. The measured transmission coefficients are -30 dB and -31 dB for the lower and higher operating ISM frequencies, respectively.

The measured AR of the designed antenna are 0.38 dB, and 1.01 dB at 2.41 GHz and 5.81 GHz, respectively.

## Conclusion

In this research, implanted Tx antenna and on-body Rx antenna with wide bandwidths in (2.41 and 5.81) GHz ISM bands were reported for wireless bio-telemetry applications. A compact dual-band CP implantable patch antenna was designed and analyzed in a three-layer phantom as well as HSM. Compared to the previous literature designs, small size, wide impedance BW, large AR BW, and good impedance matching are achieved. Furthermore, for implantable communication, a flexible on-body antenna with a compact size was introduced. The antenna was designed on a flexible substrate material for patient comfort. The performance of the antenna was studied by placing it on a three-layer model. Then, for simulation results validation, the transmission and reflection coefficients of two antennas were measured. The printed implantable antenna was implanted in a chicken slab as well as a saline solution with electrical properties mimicking the human body, and the receiving antenna was placed on the chicken slab for reflection coefficient measurements. The measured impedance BWs of the implantable antenna are 13.04 % and 33.2 % in the chicken slab, while 19.5% and 25.2 % in the saline solution at the two ISM bands, respectively. While, the measured impedance BWs of the on-body antenna mounted on the chicken slab are 24 % and 50.4 % at two operating frequencies, respectively. Then, the transmission coefficient is performed by immersing the implantable antenna in a chicken slab and placing the on-body antenna on it. There is good agreement between the measured and simulated results. For future directions of this research, implantable and on-body antennas can be integrated into IMDs and wearable devices.

## Data Availability

All data generated or analysed during this study are included in this published article.
